# Genetic Variation Bias toward Noncoding Regions and Secreted Proteins in the Rice Blast Fungus Magnaporthe oryzae

**DOI:** 10.1128/mSystems.00346-20

**Published:** 2020-06-30

**Authors:** Zhenhui Zhong, Meilian Chen, Lianyu Lin, Ruiqi Chen, Dan Liu, Justice Norvienyeku, Huakun Zheng, Zonghua Wang

**Affiliations:** a State Key Laboratory of Ecological Pest Control for Fujian and Taiwan Crops, College of Plant Protection, Fujian Agriculture and Forestry University, Fuzhou, China; b Institute of Oceanography, Minjiang University, Fuzhou, China; c College of Life Science, Fujian Agriculture and Forestry University, Fuzhou, China; USDA-Agricultural Research Service, Boyce Thompson Institute, Cornell University

**Keywords:** *Magnaporthe*, experimental evolution, genetic variant

## Abstract

The genomes of plant pathogens are highly variable and plastic. Pathogen gene repertoires change quickly with the plant environment, which results in a rapid loss of plant resistance shortly after the pathogen emerges in the field. Extensive studies have evaluated natural pathogen populations to understand their evolutionary effects; however, the number of studies that have examined the dynamic processes of the mutation and adaptation of plant pathogens to host plants remains limited. Here, we applied experimental evolution and high-throughput pool sequencing to Magnaporthe oryzae, a fungal pathogen that causes massive losses in rice production, to observe the evolution of genome variation. We found that mutations, including single-nucleotide variants (SNVs), insertions and deletions (indels), and transposable element (TE) insertions, accumulated very rapidly throughout the genome of *M. oryzae* during sequential plant inoculation and preferentially in noncoding regions, while such mutations were not frequently found in coding regions. However, we also observed that new TE insertions accumulated with time and preferentially accumulated at the proximal region of secreted protein (SP) coding genes in *M. oryzae* populations. Taken together, these results revealed a bias in genetic variation toward noncoding regions and SP genes in *M. oryzae* and may contribute to the rapid adaptive evolution of the blast fungal effectors under host selection.

**IMPORTANCE** Plants “lose” resistance toward pathogens shortly after their widespread emergence in the field because plant pathogens mutate and adapt rapidly under resistance selection. Thus, the rapid evolution of pathogens is a serious threat to plant health. Extensive studies have evaluated natural pathogen populations to understand their evolutionary effects; however, the study of the dynamic processes of the mutation and adaptation of plant pathogens to host plants remains limited. Here, by performing an experimental evolution study, we found a bias in genetic variation toward noncoding regions and SPs in the rice blast fungus Magnaporthe oryzae, which explains the ability of the rice blast fungus to maintain high virulence variation to overcome rice resistance in the field.

## Abstract (precis)

Plants “lose” resistance toward pathogens shortly after their widespread emergence in the field because plant pathogens mutate and adapt rapidly under resistance selection. Thus, the rapid evolution of pathogens is a serious threat to plant health. Extensive studies have evaluated natural pathogen populations to understand their evolutionary effects; however, the study of the dynamic processes of the mutation and adaptation of plant pathogens to host plants remains limited. Here, by performing an experimental evolution study, we found a bias in genetic variation toward noncoding regions and SPs in the rice blast fungus Magnaporthe oryzae, which explains the ability of the rice blast fungus to maintain high virulence variation to overcome rice resistance in the field.

## INTRODUCTION

Genome plasticity, mediated in part by deletions or insertions promoted by transposable elements (TEs), contributes to the adaptation of fungal pathogens to their hosts (1, 2), which renders fungal pathogens great threats to human health and food security. As with the genomes of other pathogens, the genomes of filamentous fungi encode an arsenal of effectors that play critical roles in the interaction between fungi and their hosts. Fungal effectors are initially secreted to surmount their host’s defenses, while some of them (called avirulence [*Avr*] genes) can be recognized by host R proteins and cause an avirulence phenotype (3). Most of the effectors identified so far are small secreted proteins (SPs) (4). Surveys of the sequenced genomes of filamentous fungi have indicated that fungal effector genes are not evenly distributed throughout the genome but are enriched in gene-sparse and repeat-rich two-speed genome regions where the genes undergo variation more rapidly than the genes in gene-rich compartments (2, 5, 6).

In the evolution of pathogens, mutations accumulate throughout the genome, while beneficial mutations gradually become fixed and dominant in the population. The fixation of beneficial mutations is highly dramatic and rapid in pathogens under constant selection, for instance, in a highly uniform agroecosystem (7, 8). However, the mechanisms of the evolution of pathogens in the coevolution process and the fixation of beneficial mutations in a population under the constant selection of host plants are still largely unknown. Unraveling the dynamic variations in a plant pathogen’s population while interacting with its host can enhance our understanding of pathogen evolution in nature, especially in agroecosystems, and thus provide efficient and environmentally friendly disease management strategies (9).

Many efforts have been made to uncover the dynamic variation in plant pathogen populations; among these efforts, experimental evolution has been well established in microbial population research and has become a powerful tool that is complementary to modern genetic and pathogen epidemiology research (10–14). Compared with population genetics, which ultimately provides information regarding evolution in the context of many undetectable environmental factors, experimental evolution methods can simulate the evolutionary process of organisms under controlled conditions and retain retraceable samples of different stages. The combination of well-developed and inexpensive sequencing technology enables the experimental evolution method to reveal molecular mechanisms under gradual adaptation and provide a real-time perspective of evolution dynamics.

Magnaporthe oryzae (syn. Pyricularia oryzae), the causative agent of rice blast disease, leads to a 10% to 30% reduction in rice production annually (15). Investigations have shown that *Magnaporthe* species are also capable of causing blast disease in more than 50 plant species of monocot origin in addition to rice, including food crops such as wheat, millet, and barley. In addition, *Magnaporthe* species also infect wild grass hosts such as Digitaria sanguinalis, Setaria viridis, and Eleusine indica (16). A previous study identified a novel avirulent gene, *AvrPi9*, in *M. oryzae* via the sequential planting method, which operates under a theory similar to that of experimental evolution (17, 18). In a previous study, the experimental evolution of *M. oryzae* on artificial medium resulted in a rapid accumulation of mutations prior to the observation of a phenotype; however, the results of that study also revealed a significant reduction in the virulence of the progeny population, which may be due to the lack of an infection stage (19). The genome sequence of *M. oryzae* varied greatly when it adapted to hosts from different species and subspecies of rice (20, 21). Although sequential inoculation *in planta* was performed using a mixture of *M. oryzae* isolates (17, 18), no real-time evolution studies have been conducted *in planta* so far to investigate the role of either host selection or clonality in the rapid evolution of rice blast fungus.

Next-generation sequencing of pooled samples (Pool-Seq) has been used as a cost- and time-effective approach for studying population variability and differentiation (22). In this study, we performed an experimental evolution study by embracing the advantages of the experimental evolution method and high-throughput Pool-Seq technology to monitor the coevolution process by serially inoculating pathogens into host plants to evaluate the gradual variation in *M. oryzae* in rice. We found the rapid accumulation of low-frequency single-nucleotide variants (SNVs), insertions and deletions (indels), and TEs during infection, and interestingly, these mutations were enriched in intergenic regions and the proximal region of SP coding genes, whereas they were depleted in coding regions.

## RESULTS

### Experimental evolution via sequential infection with *M. oryzae*.

**FIG 1:**
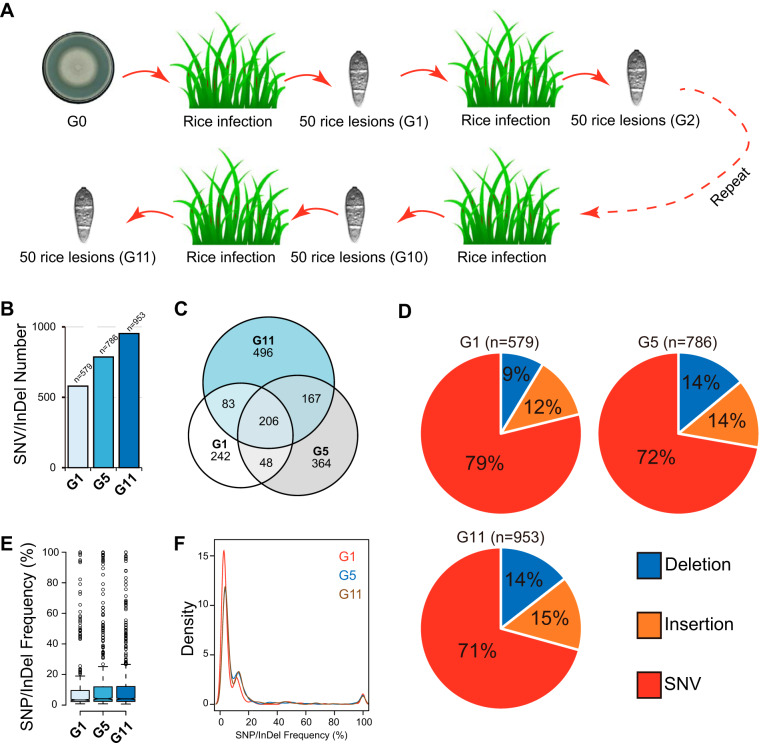
Experimental evolution of *M. oryzae*. (A) Schematic map of the experimental evolution assay used in this study. The wild-type strain Guy11 was grown on rice bran medium to generate spores that were adopted as the initial generation (G_0_). Seedlings of the susceptible rice cultivar (TP309) were inoculated with spores obtained from G_0_ to commence the first infection cycle. In total, 50 compatible leaf lesions from the first infection cycle were randomly collected, sterilized, and then incubated for 7 days on rice bran medium at 26°C under constant light to yield enough G_1_ spores. The G_1_ spores were used as the inoculum for the next infection cycle. A total of 11 infection cycles were carried out in this study to generate 11 generations of *M. oryzae* populations (G_1_ to G_11_). (B) Total number of SNVs/indels identified in G_1_, G_5_, and G_11_ Pool-Seq data. (C) Venn diagram showing overlapping of SNVs/indels identified in G_1_, G_5_, and G_11_. (D) Percentage of different types (deletion, insertion, or SNV) of SNVs/indels identified in G_1_, G_5_, and G_11_. (E) Box plot showing frequency of all SNVs/indels identified in G_1_, G_5_, and G_11_. (F) Density plot showing density distribution of SNVs/indels identified in G_1_, G_5_, and G_11_. The red line represents the G_1_ population, the blue line represents the G_5_ population, and the brown line represents the G_11_ population.

To evaluate the host-induced adaptive evolution among succeeding generations of *M. oryzae* populations, the experimental evolution assay was conducted as shown in Fig. 1A. The wild-type strain Guy11 was grown on rice bran medium to generate spores that were adopted as the initial generation (G_0_ [G stands for generation herein]). Seedlings of a susceptible rice cultivar (TP309) were spray inoculated with spores obtained from G_0_ to commence the first infection cycle (infection cycle refers to 7 days postinoculation). In total, 50 compatible leaf lesions from multiple plants of the first infection cycle were randomly collected, sterilized, and then incubated for 7 days on rice bran medium at 26°C under constant light to yield enough G_1_ spores. The G_1_ spores were used as the inoculum for the next infection cycle. A total of 11 infection cycles were carried out in this study to generate 11 generations of *M. oryzae* populations (G_1_ to G_11_).

### High-throughput pool sequencing of experimental evolution populations.

To investigate the variation in *M. oryzae* that occurred under constant host selection, whole-genome sequencing was performed using Illumina paired-end sequencing. During the evolution process, mutations occurred in different sites in each individual; however, only beneficial mutations facilitating host adaptation gradually accumulated in the population. We thus selected G_1_, G_5_, and G_11_ for in-depth whole-genome sequencing. To minimize the difference between the reference genome and the original isolate (G_0_) used in this study, the original isolate was also sequenced and used as a control in this study. To investigate mutations at the population level, we used pool sequencing (Pool-Seq), which is a cost-effective method that pools sequences of individual DNAs. For G_1_, G_5_, and G_11_, we randomly selected 50 individual isolates for each generation and combined their DNA in equal amounts for Pool-Seq.

The obtained reads of G_0_, G_1_, G_5_, and G_11_ were mapped to the genome sequence of the *M. oryzae* 70-15 reference genome for single-nucleotide variant (SNV) and insertion and deletion (indel) analyses (see Table S1 in the supplemental material). We performed SNV/indel calling separately for all four samples, and the obtained SNVs/indels were filtered with the SNVs/indels of G_0_. Overall, we obtained 579, 786, and 953 SNVs/indels for G_1_, G_5_, and G_11_, respectively (Fig. 1B). Further analysis of overlapping SNVs/indels in G_1_, G_5_, and G_11_ showed that 206 SNVs/indels were shared by the three generations and 242, 364, 496 SNVs/indels were uniquely present in G_1_, G_5_, and G_11_, respectively (Fig. 1C). To evaluate variation sites at the population level, we analyzed the SNV/indel frequency of each site in G_1_, G_5_ and G_11_ (Fig. 1F). The results showed that G_5_ and G_11_ had similar average SNV/indel frequencies (Fig. 1D and E), while G_1_ had more low-frequency sites than G_5_ and G_11_.

10.1128/mSystems.00346-20.2 TABLE S1 High-throughput pool sequencing of experimental evolution samples. Download Table S1, XLSX file, 0.01 MB. Copyright © 2020 Zhong et al. 2020 Zhong et al. This content is distributed under the terms of the Creative Commons Attribution 4.0 International license.

### Genomic distribution of SNVs/indels.

**FIG 2:**
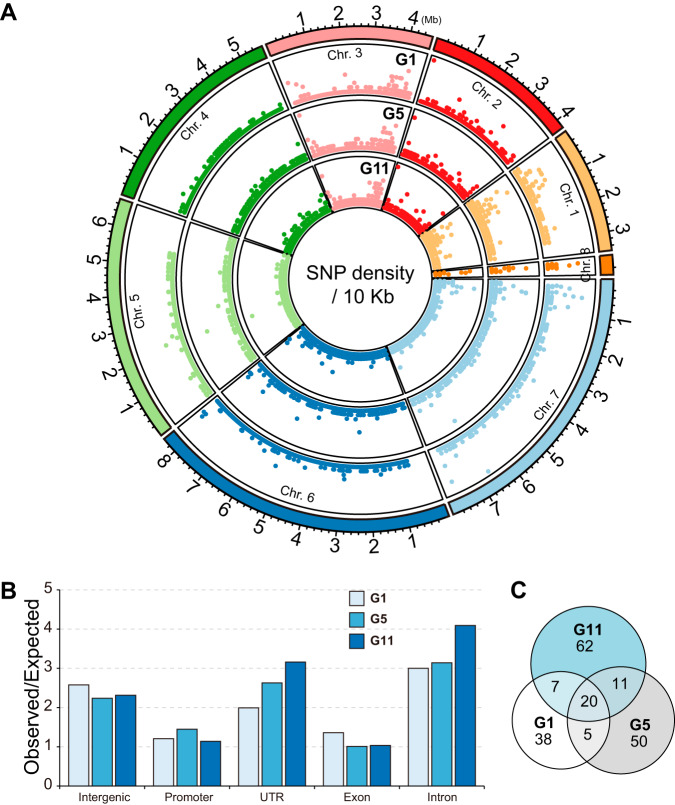
Annotation of SNVs/indels identified in the experimental evolution assay. (A) Chromosomal distribution of SNVs/indels. The numbers of SNVs/indels in 10-kb windows were calculated and plotted. (B) Genomic distribution of SNVs/indels identified in G_1_, G_5_, and G_11_. The number was calculated by comparing the expected number of SNVs/indels in each of five categories (intergenic, promoter, exon, intron, and UTR) with the observed number of sites in the genome. (C) Venn diagram showing overlapping SNV/indel-affected genes in G_1_, G_5_, and G_11_. Only frameshift variants, in-frame deletions, in-frame insertions, missense variants, start codon losses, stop codon losses, and stop codon gains that changed amino acid sequences were analyzed.

To evaluate the impact of constant selection on the pathogen’s genome, we calculated the SNV/indel number in every 10-kb region and observed that the three samples exhibited significant differences in SNV/indel distribution pattern (Fig. 2A). We found the most variation in chromosome 1 (Chr 1), Chr 3, Chr 6, and Chr 7 in the G_1_, G_5_, and G_11_ genomes, and the number of SNVs/indels increased consistently in some regions. Consistent with our previous findings that G_1_ was more heterozygous and had more low-frequency sites than G_5_ and G_11_, the distribution of SNVs/indels in G_1_ was more diversified than that in G_5_ and G_11_.

We then questioned whether these SNVs/indels had any bias in genome distribution. To answer this question, we annotated the genomic distribution of SNVs/indels by dividing the genome into five categories: intergenic, promoter (−1,000 to 0 bp), exon, intron, and 5′ and 3′ untranslated regions (UTRs). We calculated the expected numbers of SNVs/indels in these five categories by randomly selecting an equal number of control sites in the genome and comparing it with the SNVs/indels of G_1_, G_5_, and G_11_ (observed number). Interestingly, we found that the ratios of observed number/expected number of SNVs/indels were much higher in intergenic (2.58, 2.24, and 2.32) and intron regions (3.00, 3.14, and 4.09) than in exon regions (1.36, 1.01, and 1.03) in G_1_, G_5_ and G_11_, respectively, suggesting that SNVs/indels were preferentially enriched in intergenic and intron regions than in exon regions (Fig. 2B). In addition, the ratios of observed number/expected number of SNVs/indels in the promoter and exon were close to 1, suggesting no bias in distribution in these regions.

To determine the effects of SNVs/indels on genes, transcripts, protein sequences, and regulatory regions, we next investigated the effect of these SNVs/indels on gene products. Consistent with the SNV/indel distribution results, most of the SNVs/indels that caused upstream or downstream gene variations had no significant effect on gene products. Some variation types identified in this study lead to amino acid changes, including frameshift variants, in-frame deletions, in-frame insertions, missense variants, start codon losses, stop codon losses, and stop codon gains (Table 1). We found that SNVs/indels with these variations occurred in 193 genes, among which 20 were shared by 3 samples and 38, 50, and 62 were presented uniquely in G_1_, G_5_, and G_11_, respectively (Fig. 2C). Functional annotation indicated multiple functions of these 193 genes, involved in pathways ranging from the metabolic pathway and transcription regulation to the mating signal transduction pathway (Table S2). Overall, although SNVs/indels accumulated rapidly in the genome, it appeared that coding regions were not a hot spot of variation.

**TABLE 1:** Quantification of different types of effects from SNV/indel mutations

Effect type	No. of effects in:		
	G_1_	G_5_	G_11_
Splice acceptor variant	3	2	4
Stop gained	9	3	4
Frameshift variant	5	10	9
Start lost	0	0	1
In-frame insertion	1	1	1
In-frame deletion	2	1	3
Missense variant	83	101	116
Splice region variant	6	7	8
Synonymous variant	40	50	56
5′ UTR variant	52	68	90
3′ UTR variant	43	66	85
Noncoding transcript exon variant	3	4	3
Intron variant	46	83	97
Upstream gene variant	258	351	424
Downstream gene variant	28	36	51

10.1128/mSystems.00346-20.3 TABLE S2 Functional prediction and annotation of mutated genes. Download Table S2, XLSX file, 0.02 MB. Copyright © 2020 Zhong et al. 2020 Zhong et al. This content is distributed under the terms of the Creative Commons Attribution 4.0 International license.

### Dynamics of TEs in experimental evolution populations.

**FIG 3:**
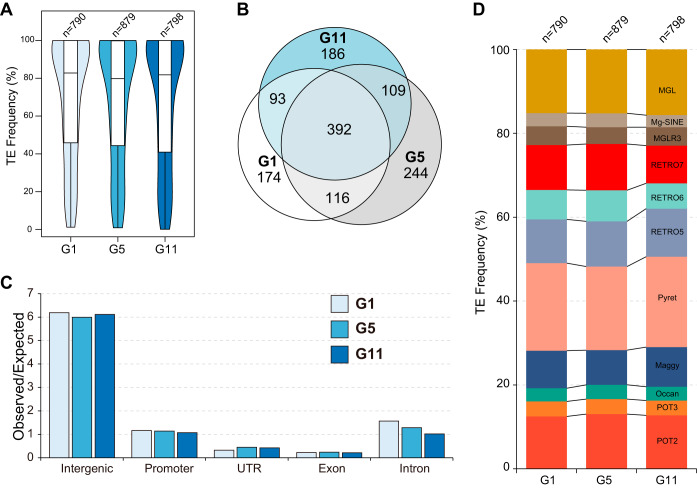
Annotation of TEs identified in the experimental evolution assay. (A) Copy number and frequency of SNVs/indels identified in G_1_, G_5_, and G_11_. The *P* value was calculated using Student's *t* test. (B) Venn diagram showing overlapping TEs identified in G_1_, G_5_, and G_11_. (C) Genomic distribution of TEs identified in G_1_, G_5_, and G_11_. The number was calculated by comparing the expected number of TEs in each of five categories (intergenic, promoter, exon, intron, and UTR) with the observed number of sites in the genome. (D) Proportion of different types of TEs among newly formed TEs in G_1_, G_5_, and G_11_.

Transposable elements (TEs) play important roles in genome shaping and genome stability in *M. oryzae* (23, 24). Plant pathogens can avoid plant host immunity recognition through TE insertion-mediated silencing of some avirulent genes (25, 26). We thus set out to analyze TE dynamics in experimental evolution populations. We first annotated newly formed TE insertion events in G_1_, G_5_, and G_11_ by annotating all TE insertion events in the genome and filtering them with G_0_. We identified 790, 879, and 798 newly formed TE insertion events in G_1_, G_5_, and G_11_, respectively (Fig. 3A). A Venn diagram showing the overlapping TEs in the three samples indicated that more than 50% of TEs were shared among these samples and that 186 TEs were uniquely present in G_11_ (Fig. 3B). The insertion of TEs at the promoter region results in gene silencing; thus, we investigated the relationship between the TE insertion site and the coding gene. We found that, as observed in the SNV/indel analysis, new TEs were preferentially enriched in intergenic regions and depleted in exon regions (Fig. 3C). To determine the activity of these TEs, we calculated the copy number of newly formed TEs in G_1_ (*n* = 790), G_5_ (*n* = 879), and G_11_ (*n* = 798). In general, long-terminal-repeat (LTR) retrotransposons (Maggy, RETRO5, RETRO6, RETRO7, Pyret, and MGLR3) are more active than DNA transposons (POT2, POT3, and Occan). We found that among the LTR retrotransposons, Pyret was the most active TE, followed by long interspersed elements and MGL (Fig. 3D). Compared with POT3 and Occan, POT2 was the most active DNA transposon. In summary, extensive variation in TE copy number was observed in the process of successive host interaction, and TEs were preferentially enriched in intergenic regions and depleted in exon regions.

### TE insertion in genes encoding SPs.

**FIG 4:**
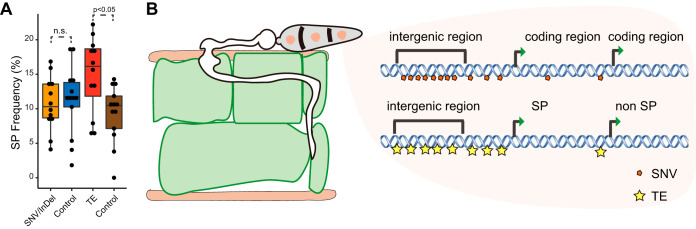
Bias in transposable element (TE) insertion in secreted protein (SP) coding genes. (A) Percentage of SP genes among genes with newly formed TE insertions. Dots in the box plot include promoter, exon, intron, or TTS region data. The *P* value was calculated using Student's *t* test. Detailed data for each dot are shown in Fig. S1 in the supplemental material. n.s., nonsignificant. (B) Proposed model depicting the changes in an *in planta* experimental evolution assay of *M. oryzae*. Single-nucleotide variants (SNVs) and TE insertions accumulated rapidly throughout the genome of *M. oryzae* in noncoding regions. TE insertions preferentially accumulated in the promoter regions of SP coding genes in *M. oryzae*.

Secreted proteins (SPs) play dual functions in plant-pathogen interactions (27, 28). They can facilitate pathogen infection by overcoming the plant defense response, such as suppressing the plant immune system or hijacking host metabolism (29). Some SP genes (*Avr* genes) can be recognized by plant *R* gene products to induce plant immunity. Previous studies indicated that this group of genes is a hot spot for SNV variation and TE insertion because these variations can help pathogens avoid plant recognition (17, 25, 26). However, so far, there has been no direct evidence to support the preferential insertion of TEs in SP genes. Therefore, we investigated the percentage of SP genes among genes that have newly formed TE insertions in the promoter, exon, intron, or transcription termination site (TTS) regions. The abundance of SP genes in the whole genome was 11.84% (1,539/12,991), which was similar to that of SNV/indel-associated genes (10.69%), the control set of SNV/indel-associated genes (11.15%), and the control set of TE-associated genes (10.33%); however, the abundance of SP genes in the whole genome was significantly (*P* < 0.05) lower than that of TE-associated SP genes (16.32%), especially at promoter and intron regions (Fig. 4A; Fig. S1A and B). This result provides direct evidence to support the hypothesis that TEs tend to insert into SP genes.

10.1128/mSystems.00346-20.1 FIG S1 Bias in TE insertion in secreted protein (SP) coding genes. (A) Percentage of SP and non-SP genes among genes that contain SNV/indel variations. (B) Percentage of SP and non-SP genes among genes that contain newly formed TE insertions. Only genes that had SNVs/indels or TEs in the promoter, exon, intron or TTS regions were analyzed, whereas intergenic SNVs/indels or TEs were not included. Download FIG S1, PDF file, 0.4 MB. Copyright © 2020 Zhong et al. 2020 Zhong et al. This content is distributed under the terms of the Creative Commons Attribution 4.0 International license.

## DISCUSSION

Genome plasticity allows the rapid evolution of plant pathogens and enables plant pathogens to overcome host resistance quickly, which makes the control of fungal diseases challenging. The filamentous fungus *M. oryzae* is the causal pathogen of rice blast disease, which leads to substantial losses in rice production annually (15, 30). Our previous studies suggested that the host plant is the major force that shapes the *M. oryzae* genome (20, 21, 31). However, no real-time assays have been performed so far to investigate the occurrence and accumulation of mutations in *M. oryzae* populations in interaction with rice plants. To this end, we performed the *in planta* experimental evolution of *M. oryzae* by monitoring the genomic variation in serially subinoculated populations.

During adaptation to an alternative host, pathogens are thought to maintain a balance between generalism and specialization and finally to reach a suboptimal fitness (32, 33). In this study, by monitoring the genomic variation in a population of progeny derived from sequentially inoculating *M. oryzae*, we found that G_1_ presented a vast number of variations and that a large number of variations were shared by all samples. These results suggested that genomic variations occurred rapidly within a very short period of time and accumulated very rapidly in the genome and that G_1_ has a founder effect on the sequential inoculation population. Because TP309 is not the original host of the Guy11 strain, it may possess immunity to Guy11, in contrast to its original host plant. We therefore proposed that the fungal genome may have been subjected to strong selection in response to plant immunity at the beginning of the experiment and reached a bottleneck when the strain showed optimal fitness under continuous selection in this plant variety. Similar results were shown by Jeon et al. (19). However, the host plant may impose a much more specific and efficient selection on the fungal genome than does oatmeal medium. In the future, using strains to inoculate their original host plants might further illustrate whether such bottleneck effects are common during adaptive evolution. It is also worth noting that although the medium cultivation process has been minimized and mimics the natural rice environments by using rice bran medium to exclude most of the selection stress on the isolates in this study, the medium cultivation process may still result in some stress on the population that is different from host selection, resulting in some abiotic stress variations in the *M. oryzae* genome. Development of advanced sequencing technologies, such as single-cell sequencing, may provide resolution to waive the artificial effects caused by medium culture. Taking these results together, we propose that *M. oryzae* maintained a balance between population diversity and bottleneck effects while adapting to its host plant.

Notably, genetic variation does not neutrally accumulate in the genome during adaptive evolution. We observed that increased mutations accumulated in Chr 1, Chr 3, Chr 6, and Chr 7, and this result is consistent with that of a previous experimental evolution study (19). It is possible that there are mutation hot spots in these chromosomes, or alternatively, these chromosomes might buffer more mutations and undergo stronger selection. Interestingly, more SNV/indel mutations were found in noncoding regions than in coding regions, as shown previously by Jeon et al. (19). The different mutation rates in the noncoding (intergenic) and coding (exon) regions can at least partially explain the accumulation of variations in *M. oryzae* in a short period of time despite *M. oryzae* rarely showing phenotypic variations. We proposed that this bias indicates a purifying selection on coding regions, especially some important housekeeping genes, to maintain genome stability. To search for genes that are involved in the adaptive process, we annotated the genomic consequences of these variations and found that these genes were involved in multiple pathways.

Previous studies have indicated that transposable elements (TEs) play important roles in host-pathogen interactions. TEs are major components of facultative heterochromatin regions that provide not only epigenetic regulation of the transcription of effector/secreted protein (SP) genes but also a cradle for rapid adaptive evolution (6, 34) and can induce total silencing of *Avr* genes and thus help pathogens avoid host recognition (25, 26, 35). Our results showed that G_1_ also has a founder effect on newly formed TE insertion, although the number of new TE insertions gradually increased in G_5_ and G_11_. These results suggested that the transposability of TEs in the G_1_ fungal genome is the highest. In line with the bias mutation rate of SNVs/indels in the intergenic and exon regions, TE insertions were also enriched in the intergenic region and depleted in the exon region. In addition, we found that Pyret was the most active TE. This result is supported by a previous study, which also found that Pyret was the most active TE under different stresses (36).

The pathogen effector/SP genes play pivotal roles during the interaction with their host, especially for biotrophic or hemibiotrophic pathogens (27, 28). These genes are enriched preferentially in repeat-rich regions and are thought to be evolved more rapidly than genes in other genomic compartments (17, 25, 26). Moreover, most of these genes are functionally redundant (37) and could tolerate more mutations, which provides genetic diversity for adaptive evolution on diverse hosts. In *M. oryzae*, an increasing amount of evidence has demonstrated that SP genes, especially the *Avr* genes, tend to evolve more rapidly than other genes. For instance, comparative genomic analysis revealed that the SP genes presented a high level of diversity among different strains (38–40). In addition, some of the SP genes (*Avr* genes) have been suggested to be hot spots for SNV variation and TE insertion as a tool for avoiding plant recognition (17, 25, 26, 41). Consistent with this hypothesis, we found that in addition to the intergenic region, TEs also showed an insertion bias toward genes encoding SPs. These results suggested that SP genes are very important for adaptive evolution toward diverse rice hosts (different subspecies/cultivars) and that TEs are one of the major forces fueling the rapid evolution of *M. oryzae*. The high bias toward TE insertion into SP genes indicated that TE insertion at the *Avr* gene may not be neutral.

In conclusion, we applied an experimental evolution assay to *M. oryzae* and identified the preferential accumulation of SNVs/indels and TEs in noncoding regions and SP genes (Fig. 4B). Further studies on the genetic function of the mutated genes identified in this study will provide more insights into the adaptive evolution of *M. oryzae*. We believe that the results obtained through experimental evolution may also enhance our understanding of pathogen evolution in nature and explain the mechanism of the vulnerability of rice resistance.

## MATERIALS AND METHODS

### Evolution experiment assay.

*Magnathorpe oryzae* strain Guy11, isolated from French Guyana (42), was used as the initial strain in this study. Single spores isolated from diseased rice (TP309, a cultivar susceptible to Guy11) leaves 7 days postinoculation (dpi) of each generation were grown on rice bran medium (2% rice polish and 1.5% agar at pH 6.5) at 26°C under constant light for conidiation. Small pieces of sterilized filter paper were also put on the medium and collected when it was colonized by the fungus. The fungal cultures on the dried filter paper were then stored in a –80°C refrigerator. Genomic DNA samples were extracted using the CTAB extraction method from mycelia cultured in liquid CM medium (0.6% yeast extract, 0.6% casein hydrolysate, 1% sucrose, and 1.5% agar) with 130 rpm shaking at 26°C for 3 to 4 days. Detailed steps for the experimental evolution assay are described in Results and in the legend for Fig. 1A.

### Genome sequencing.

DNA samples from individual isolates were combined in equal amounts for sequencing. DNA samples were sheared to ∼350 bp in average size. Sequencing libraries were prepared using the Illumina paired-end DNA sample prep kit and sequenced on an Illumina HiSeq 2500 platform with 2 × 150-bp paired-end reads.

### Read alignment and SNV/indel calling and annotation.

SNV/indel calling was performed according to a previously described method with some modifications (43, 44). Briefly, all sequenced reads were aligned to the *M. oryzae* 70-15 reference genome with Bowtie2 with the default parameters (45). The resulting bam files were subjected to Picard MarkDuplicates function to remove PCR duplicates (https://broadinstitute.github.io/picard). PCR-duplicated reads were removed, and the remaining files were then subjected to variant calling. Additionally, mapping reads with a mapping quality (MAPQ) value of >40 were retained for variant calling. CRISP has been used for variant calling with a minimum of 10 reads with alternate alleles (46). The SNV/indel density was calculated by VCFtools (v0.1.15) in every 10-kb window and visualized with circlize (47, 48). The SNV/indel control data set was randomly selected from the whole genome with BEDTools (v2.21.0) (49). The genomic distribution of the SNVs/indels was annotated with ChIPseeker (v1.20.0) (50). The types of consequences of the SNVs/indels were predicted by VEP (v96) and the *Magnaporthe* 70-15 reference genome version 43 from EnsembleFungi (https://fungi.ensembl.org/Magnaporthe_oryzae/Info/Index) (51).

### TE annotation and SP prediction.

RepeatMasker (version 3.3.0; http://www.repeatmasker.org/) was used to search for TEs in 70-15 (52). The presence and absence of TE polymorphisms were detected by PoPoolationTE2 with the 70-15 sequence as the reference genome and at least 3 reads of support (53). TE insertions supported by at least 3 reads in G_0_ were maintained for TE filtering in the G_1_, G_5_, and G_11_ samples. The genomic distribution of TE insertions was annotated with the annotatePeaks function in Homer2 (v4.8.3) (54). Peaks within 50 bp between two samples were merged into the same insertion by BEDTools (v2.21.0) (49). The TE control data set was randomly selected from the whole genome with BEDTools (v2.21.0) (49). SPs were defined as proteins containing a signal peptide cleavage site, no transmembrane domain after the signal peptide cleavage site, and an amino acid sequence length smaller than 400 amino acids. SignalP 5.0 was used to predict signal peptides, and TMHMM 2.0 was used to predict transmembrane domains (55, 56).

### Statistical analysis.

All statistical analyses were performed using Student's *t* test function in R (57).

### Data availability.

All genomic sequencing data were deposited in the NCBI Sequence BioProject database under accession number PRJNA577277.
