# Supplementary figures and images for: Genetic Variation Bias toward Noncoding Regions and Secreted Proteins in the Rice Blast Fungus Magnaporthe oryzae
Source: mSystems. 2020 Jun 30;5(3):e00346-20. doi: 10.1128/mSystems.00346-20 (PMC7329325; doi:10.1128/mSystems.00346-20)

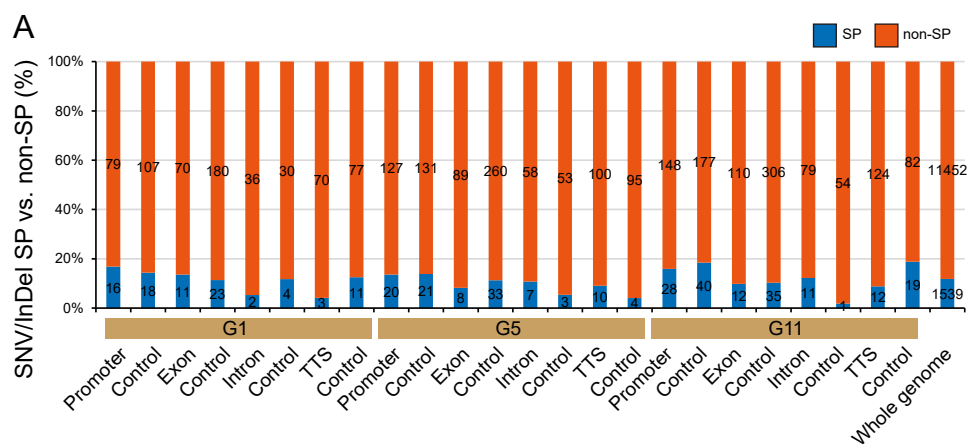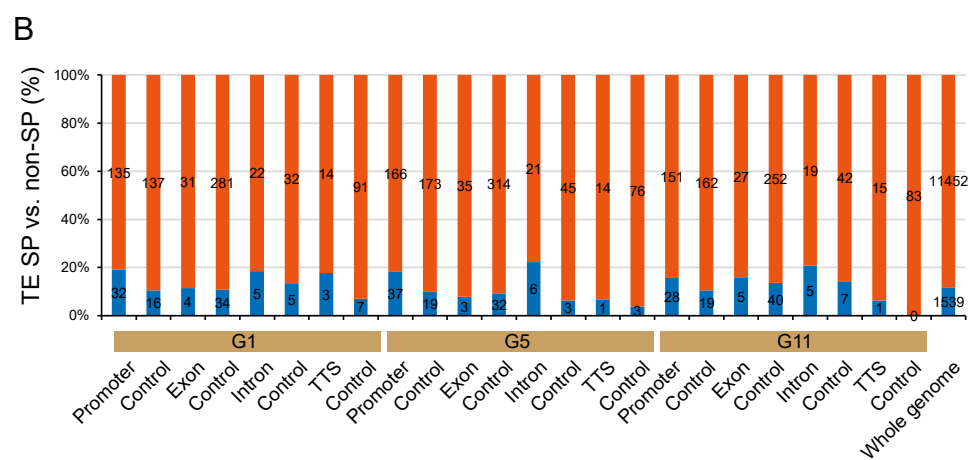

Supplement: FIG S1 [file mSystems.00346-20-sf001.pdf]
